# Features of non-stenotic carotid plaque on computed tomographic angiography in patients with embolic stroke of undetermined source

**DOI:** 10.3389/fcvm.2022.971500

**Published:** 2022-08-23

**Authors:** Danling Guo, Sangying Lv, Guanzuan Wu, Haifeng Li, Bo Wei, Jianfeng Yang

**Affiliations:** ^1^Department of Radiology, Shaoxing People's Hospital, Shaoxing, China; ^2^Department of Neurology, Shaoxing People's Hospital, Shaoxing, China

**Keywords:** embolic stroke of undetermined source, computed tomographic angiography, non-stenotic carotid plaque, plaque features, risk factor

## Abstract

**Purpose:**

Embolic stroke of undetermined source (ESUS) is a subset of cryptogenic stroke constituting a large proportion of acute ischemic strokes. This study aimed to assess the features of non-stenotic carotid plaque (<50%) on computed tomographic angiography (CTA) and to evaluate the association between non-stenotic carotid plaque and ESUS.

**Methods:**

From January 1 to December 31, 2019, a total of 60 consecutive patients with primary unilateral ESUS and <50% carotid artery stenosis, as determined using screening ultrasonography, were hospitalized in the Department of Neurology of our hospital. All enrolled patients underwent CTA to determine the composition and morphological features of non-stenotic carotid plaques using consecutive sections in both carotid arteries. The features of these plaques with and without ipsilateral stroke in patients with ESUS were compared.

**Results:**

Sixty ESUS images were included in the study, with 85 plaques. Forty-five (52.9%) of these plaques were ipsilateral and 40 (47.1%) were contralateral to the stroke. Compared to that of the contralateral plaque group, the maximum carotid plaque thickness and plaque length of the ipsilateral group were greater (2.1 mm vs. 1.5 mm, *p* = 0.03; 20.8 mm vs. 12.1 mm, *p* = 0.02); however, there were no significant differences in the degree of luminal stenosis, presence of soft plaque and calcified plaque, and the number of ulcers on the plaque surface between the two groups. Similarly, the number of plaques with thickness >3 mm in the ipsilateral group was greater than in the contralateral group (30 vs. 13, *p* = 0.01). A lipid core was more common in individuals with ipsilateral strokes than in those with contralateral strokes (19 vs. 7, *p* = 0.02). Regression analysis showed that plaque lipid core area was an independent risk factor for ESUS (odds ratio, 1.92; 95% confidence interval, 1.22–3.04; *p* = 0.03).

**Conclusions:**

Non-stenotic carotid plaques could be an etiology of acute ischemic strokes classified as ESUS. The presence of a lipid core was a risk factor in individuals with non-stenotic carotid plaques.

## Introduction

Acute ischemic stroke is a leading cause of death worldwide (1). Early identification of the possible etiology is of great importance for the prevention of future strokes. Despite a detailed classification system and extensive investigations, nearly one-third of acute ischemic strokes have no definite cause and are classified as embolic strokes of undetermined source (ESUS) (2). Patients with ESUS are far from being a homogeneous entity, whose clinical presentation and etiology are varied. In the view of recent hierarchical cluster analysis, three main phenotypes have been identified in ESUS patients with different underlying pathogenic mechanisms (3). The heterogeneity and often overlap of embolic sources of these three phenotypes make its treatment clinically challenging (4). It may be possible explanations for the best treatment for preventing stroke recurrence in this population has not been delineated, and different individualized treatment interventions may be needed (4). Further studies of identification of the possible etiology for ESUS are crucial.

Established clinical trials and guidelines suggest that significant stenosis (≥50%) in the proximal carotid artery is the leading cause of ischemic stroke (5, 6). Nonetheless, this approach ignores the embolization potential in mild-to-moderate (<50%) atherosclerotic disease. Several studies have revealed that certain morphological features of carotid plaques on magnetic resonance imaging (MRI) are independent risk factors for stroke, regardless of the degree of stenosis (7, 8). According to a recent study, <50% carotid stenosis with high-risk plaque features was significantly more common in ESUS ipsilateral to the stroke (9). As such, there has been a recent interest in high-risk non-stenotic plaques as a possible mechanism for a larger proportion of strokes, particularly ESUS.

Although MRI enables the detailed identification of vulnerable plaque features, it has poor availability and high costs. Computed tomographic angiography (CTA) is a routine, easy-to-implement, and high-resolution technique that can also offer detailed characterization of carotid plaque composition. It is now a first-line non-invasive imaging investigation for patients with acute ischemic stroke. Several studies on carotid plaques have focused on morphological features, such as degree of stenosis or plaque size (10, 11). Despite recent progress, the association between ESUS and non-stenotic carotid plaque components on CTA remains unclear. Therefore, we hypothesized that non-stenotic carotid plaque features on CTA are associated with an increased risk of ipsilateral stroke in this patient population. This study aimed to evaluate the internal morphological features and components of non-stenotic carotid plaques using CTA technology in patients with anterior circulation ESUS. We sought to explore the correlation between non-stenotic carotid plaque and the occurrence of ESUS and to identify high-risk plaque features that independently predict ESUS.

## Materials and methods

### Study population and inclusion criteria

This study retrospectively analyzed consecutive patients hospitalized in the Department of Neurology of our hospital, from January 1, 2019, to December 31, 2019, due to an initial acute unilateral ischemic stroke. Within our cohort, we identified patients with a carotid territory infarction who met the recently proposed diagnostic criteria for ESUS (2).

All patients visited a doctor within 7 days of onset of ischemic stroke. Within 10 days of hospital admission, all patients underwent CTA examination after cervical vascular color Doppler ultrasound screening revealed extracranial internal carotid artery (ICA) stenosis <50%. Patients completed the necessary diagnostic protocol for ESUS during hospitalization, including brain CT or magnetic resonance imaging within 2 weeks of onset, imaging of the cervical and intracranial arteries, electrocardiographic monitoring for at least 48 h, 24-h Holter electrocardiography, transcranial color-coded sonography screening for right-to-left shunt, and transthoracic echocardiography.

The exclusion criteria were: (1) extracranial ICA stenosis ≥50%; (2) acute stroke with bilateral or multi-vascular lacunar infarction; (3) identifiable cardioembolic source, such as atrial fibrillation, left atrial thrombosis, or recent myocardial infarction; and (4) stroke with other determined etiology, such as atherosclerotic embolism, cancer, arteritis, or vasospasm.

### CTA

Imaging was performed using a Canon Aquilion ONE (Toshiba Medical Systems, Tokyo, Japan), equipped with 320 detector rows, each 0.5 mm in width. The scanning range was 2-3 cm, from the lower edge of the aortic arch to the top of the skull. The scan was helical with a 1.0 mm section thickness (120 kV, 300 mA), gantry speed 0.625 s/rotation, and table speed 25.5 mm/rotation. Images were reconstructed in the axial plane with 0.5 mm slice thickness. The intravenous iodinated contrast was iohexol (370 mg/mL; Omnipaque, GE Healthcare, Shanghai); 70 mL was injected at 5 mL/s. Acquisition was triggered automatically by an attenuation of 100 Hounsfield units (HU) in the aortic arch.

### Image analysis

Images were analyzed using Vitrea 4.0 (Canon Medical Informatics, Minnetonka, MN, USA), a semi-automated post-processing software. Each CTA image was reviewed by one of two neuroradiologists with 5 years of experience who was blinded to the imaging and all other clinical information at all times. The neuroradiologist identified the inner and outer walls of the blood vessels and the plaque margin by adjusting the window and level settings. The carotid artery was assessed from 2 cm proximal to 2 cm distal to the bifurcation to determine the degree of stenosis on each side, using the NASCET criteria (North American Symptomatic Carotid Endarterectomy Trial). Combining the source axial and sagittal images, the maximum plaque thickness was approximated perpendicular to the long axis of the vessel in the narrowest portion of the carotid artery.

Categorical carotid plaque characteristics on CTA included the presence of a lipid core (present in at least one slice) and lipid, calcific, and fibrotic components. After drawing the region of interest of the carotid plaque, different compositions of plaques were automatically identified by post-processing software, with three pseudo-colors: lipid components were expressed in red, with CT values ranging from −100 to 30 HU; fibrous components were expressed in blue, with CT values ranging from 50 to 150 HU; calcific components were expressed in yellow, with CT values ranging from 150 to 350 HU and from 350 to 1300 HU, respectively. The lumen of the carotid arteries were expressed in green. In addition, the software automatically calculated the area of different plaque compositions at each slice, expressed in square millimeters (mm^2^). Continuous measures of carotid plaque characteristics included maximum lipid core area, total lipid core area, total calcific area, and total fibrotic area, in addition to the areas of each slice. If the artery wall was not visible or its measured diameter was < 1.0 mm, the artery was scored as plaque-free. An ulcerated plaque was defined as focal contrast extending beyond the vascular lumen by >1 mm in depth, with a well-defined back wall at the base. The observer measured the total plaque length from its proximal to its distal end, parallel to the long axis of the vessel, in millimeters (out to 1 decimal point).

To evaluate inter-observer variability, all patient results were subjected to blind review and confirmed by a second neuroradiologist using the same technique and in the same manner as the first reader.

### Statistical methods

We compared baseline demographic and plaque-associated variables between patients with ipsilateral and contralateral strokes. Continuous variables were analyzed using an independent-sample *t*-test. Categorical variables were analyzed using the χ^2^ or Fisher's exact test. Categorical variables are presented as absolute and relative frequencies, and continuous variables are presented as means ± SD. Univariate and multivariate logistic regression analyses were performed to identify plaque features associated with ipsilateral ESUS and their odds ratios (OR). All analyses were performed using SPSS version 19 (IBM Corp., Armonk, NY, USA), and *p*-values <0.05 were considered statistically significant.

### Standard protocol approvals, registrations, and patient consents

This study was approved by the Research Ethics Board of our hospital.

## Results

A total of 1,538 patients were admitted for acute ischemic stroke during the study period. We enrolled 60 patients who met the inclusion and exclusion criteria. Each was allocated into an ipsilateral ischemic stroke group (ipsilateral group) or a contralateral ischemic stroke group (contralateral group), according to whether the extracranial non-stenotic ICA plaque was ipsilateral to the blood vessel supplying the infarcted area. Among them, 28 patients were in ipsilateral group and 32 were in contralateral group. In patients with ESUS, 85 non-stenotic atherosclerotic plaques were found, which were more commonly ipsilateral than contralateral to the ischemic stroke (45/85 ipsilateral vs. 40/85 contralateral). The baseline characteristics and medical histories of the patients are shown in Table 1.

**Table 1:** Baseline characteristics of all ESUS patients [$\bar{x}$ ± s or *n*(%)].

**Variable**	**All patients**	**Ipsilateral group**	**Contralateral group**	***P*-value**
	**(*n* = 60)**	**(*n* = 28)**	**(*n* = 32)**	
Male (%)	36 (60)	20 (33.3)	16 (26.7)	0.08
BMI	24.2 ± 3.5	24.20 ± 3.9	24.20 ± 3.6	0.35
Age (years)	65.4 ± 10.5	67.4 ± 10.3	63.2 ± 10.8	0.88
Medical history, *n* (%)				
Hypertension	30 (50)	14 (23.3)	16 (26.7)	0.60
Diabetes mellitus	16 (26.7)	10 (20)	6 (6.7)	0.16
Smoking	14 (23.3)	8 (20)	6 (3.3)	0.54
Drinking alcohol	12 (20)	6 (10)	6 (10)	0.52
Hyperlipidemia	32 (53.3)	16 (26.7)	16 (26.7)	0.38
Coronary heart disease	10 (16.6)	2 (3.3)	8 (13.3)	0.16

### Plaque features associated with ipsilateral and contralateral ESUS

Among all 85 non-stenotic plaques, maximum carotid plaque thickness and plaque length were greater in ipsilateral ischemic strokes than in contralateral strokes (2.1 mm vs. 1.5 mm, *p* = 0.03; 20.8 mm vs. 12.1 mm, *p* = 0.02). Similarly, the number of plaques with a thickness >3 mm was greater in the ipsilateral group than in the contralateral group (30 vs. 13, *p* = 0.01). A lipid core was present in 19 individuals (22.3%), more commonly in individuals with ipsilateral stroke than in those with contralateral stroke (19 vs. 7, *p* = 0.02). The lipid core was larger in the ipsilateral group than in the contralateral group (7.6 ± 1.3 mm^2^ vs. 4.1 ± 1.2 mm^2^, *p* = 0.04). None of the other plaque features on imaging (degree of stenosis, number of calcified vs. predominantly non-calcified plaques, ulceration, plaque load) was significantly associated with ipsilateral stroke (Table 2).

**Table 2:** Imaging features of non-stenotic plaques ipsilateral vs. contralateral to the side of the stroke [$\bar{x}$ ± s or *n*(%)].

	**Total plaque number**	**Ipsilateral group**	**Contralateral group**	***P*-value**
	**(*n* = 85)**	**(*n* = 45)**	**(*n* = 40)**	
Maximum plaque thickness (mm)	1.7 ± 1.2	2.1 ± 2.1	1.4 ± 1.5	0.03
Plaque thickness >3 mm *n* (%)	43 (50,5)	30 (35.2)	13 (15.3)	0.01
Plaque length (mm)	15.3 ± 7.9	20.8 ± 8.4	12.1 ± 7.4	0.02
Maximum stenosis,%	39.1 ± 20.3	40.5 ± 15.4	36.1 ± 24.0	0.15
Lumen area	45.3 ± 7.9	30.8 ± 8.4	32.1 ± 7.4	0.23
Plaque classification *n* (%)				
Non-calcified plaque	32 (37.6)	19 (22.4)	13 (15.2)	0.52
calcified plaques	37 (43.5)	17 (20.0)	20 (23.5)	0.51
Mixe plaque number	16 (18.8)	9 (10.6)	7 (8.2)	0.65
Plaque components area, mm^2^				
Lipid area	6.3 ± 1.9	7.6 ± 1.3	4.1 ± 1.2	0.04
Calcification area	9.3 ± 2.3	8.7 ± 2.6	9.1 ± 3.2	0.12
Fiber area	7.4 ± 3.9	6.8 ± 3.4	7.1 ± 2.4	0.17
Plaque lipid core *n* (%)	26 (30.5)	19 (22.3)	7 (8.2)	0.02
Plaque load (PB)	0.25 ± 0.11	0.24 ± 0.10	0.21 ± 0.13	0.3
Plaque ulceration *n* (%)	39 (45.8)	24 (28.2)	15 (17.6)	0.19

### Analysis of risk factors for ESUS

In a multivariable logistic regression analysis, ipsilateral stroke was associated with the lipid core area [OR, 1.92 (95% confidence interval, 1.22–3.04)], but not with other plaque features (Table 3). Multivariate logistic regression analysis showed that lipid core was the most important risk factor for ESUS.

**Table 3:** Associations between plaque high-risk features and ESUS.

**Variable**	**OR (95% CI)**	***P-value***
Maximum plaque thickness (mm)	1.13 (0.92–1.44)	0.15
Plaque length (mm)	1.14 (0.66–1.97)	0.64
Maximum stenosis degree of lumen	1.29 (0.69–1.97)	0.43
Plaque thickness >3 mm	1.21 (0.76–1.92)	0.44
Lumen area	1.75 (0.85–3.60)	0.13
Plaque load	1.16 (0.62–2.16)	0.65
Ulceration	1.49 (0.82–2.70)	0.19
lipid core area	1.92 (1.22–3.04)	0.03
fiber area	1.32 (0.72–1.94)	0.33
calcification area	1.44 (0.93–2.24)	0.11

## Discussion

We retrospectively evaluated, in 60 consecutive ESUS patients, the characteristics and morphological features of non-stenotic carotid plaques, and the components of these plaques, using CTA technology. Our study demonstrated two key findings. First, we found that non-stenotic carotid plaques were closely associated with the occurrence of ESUS. Second, by comparing the plaque compositions in patients with ipsilateral and contralateral ESUS, we found that CTA holds promise for identification of the culprit non-stenotic carotid artery in patients with ESUS.

In this study, we found that non-stenotic carotid plaques were significantly more common ipsilateral than contralateral to ischemic stroke in ESUS patients with proven anterior circulation vessel occlusion. These findings suggest that non-stenotic carotid plaque is associated with patients currently classified as having ESUS; this has also been reported in several recent studies (10–12). Regarding morphological features, the thickness and length of a plaque ipsilateral to the stroke are larger than those on the contralateral side (11). In addition, our study showed that plaques with a thickness >3 mm (approximately one-third) are more commonly ipsilateral to the stroke. These results are consistent with a recent study suggesting that larger non-stenotic carotid plaque size is associated with ESUS (11). It has been suggested that large carotid artery plaque size contributes to strokes, not due to hemodynamic changes, but due to artery-artery embolization, even when carotid plaque involves <50% of the arterial lumen (13). We found no significant differences in other plaque morphological features between those with ipsilateral and contralateral ESUS. A likely reason is that the CTA plaque imaging was performed after the patient became symptomatic, and the plaque might have undergone a morphological change from its pre-stroke state (14).

Plaque size is a secondary marker of the underlying pathophysiology (10). Our study further assessed the detailed components of non-stenotic carotid plaques on CTA images to determine associations of component features with ipsilateral ESUS. We observed a hypodense region in some of the carotid plaques on CTA, suggesting a lipid core. We scored this feature as a part of this study. We found that the total lipid core area of an ipsilateral plaque was significantly larger than that of a contralateral plaque. Moreover, our study found that the number of lipid cores ipsilateral to the stroke was greater than that on the contralateral side. Our results indicate that the lipid core might be a risk factor for ESUS. This is also supported by a study reported by Kelly, suggesting that a region of high 18F-FDG uptake is consistent with the lipid-rich core detected histologically in carotid artery plaques, which is an independent predictor of early stroke in patients with carotid artery stenosis (15). In a longitudinal study of 120 asymptomatic individuals, carotid plaques with a maximum percentage of lipid-rich core > 40% were more likely to develop future cardiovascular events during 3-year follow-up, compared with individuals with a percentage of lipid-rich core <40% (16). This suggests that the larger the lipid core volume, the higher is the incidence of future stroke events. A study reported by Gupta et al. showed that the prevalence of hypodense regions is higher in patients with recent ischemic symptoms than in stable patients (17). More recently, one study confirmed that the presence of a lipid core is associated with future cardiovascular events, independent of plaque morphological features (18). These results suggest a possible association between the lipid core and ipsilateral ischemic stroke. The pathophysiology might be that the gradual increase in the lipid core size has toxic effects on cells, causing degradation of collagen in the fibrous cap and eventually leading to plaque rupture and cardiovascular events. Moreover, the continuous expansion of the lipid core causes great physical stress on the fibrous cap and finally promotes its rupture (19). At present, there is increasing evidence of a correlation between non-stenotic carotid plaques and ESUS. Our results support this hypothesis and provide additional evidence that the lipid core of the carotid plaque is a risk factor for stroke, as shown in our multivariate regression analysis. Compared to traditional cardiovascular risk factors, lipid cores improve the risk prediction for cardiovascular disease events. In our study, the association between other plaque components and ipsilateral strokes was weak and lacked statistical significance. Analysis of the nature of non-stenotic plaques revealed no correlation between soft plaques, calcified plaques, and ipsilateral infarction in patients with ESUS. This is consistent with previous studies of patients with >50% stenosis (20).

Our study had several limitations. First, we study found that lipid core is associated with a higher presence of ESUS; however other high-risk characteristics of plaque, such as fibrous cap, were not included. This is due to the overlap of Hounsfield units and low soft tissue contrast of CT, making it difficult to reliably identify certain plaque morphology, such as a fibrous cap. Automated segmentation might improve the performance of CTA in plaque characteristics analysis. Second, the definitions of ESUS stroke were slightly different in a couple of studies. Several studies included patients with a cryptogenic stroke and had a broader inclusion criteria than those including patients with ESUS only. We believe that uniform definitions of ESUS are needed for future studies. Finally, the design was retrospective, and a limited set of variables were analyzed. Prospective studies and studies with a larger sample might help to provide stronger evidence for determining a relationship between non-stenosis carotid plaque and ESUS.

In conclusion, we report a complex relationship between non-stenotic carotid plaque features and ESUS. Non-stenotic carotid plaques with a higher lipid core component were more common in ipsilateral ESUS than in contralateral ESUS. These findings suggest that CTA should be routinely performed in patients with ESUS, and prospective studies are needed to investigate the interrelation of these different plaque components on CTA with ESUS events.

## Data availability statement

The raw data supporting the conclusions of this article will be made available by the authors, without undue reservation.

## Ethics statement

The studies involving human participants were reviewed and approved by Academic Ethics Committee of Shaoxing People's Hospital. The patients/participants provided their written informed consent to participate in this study.

## Author contributions

DG performed the majority of the writing. SL and GW reviewed the literature, contributed to manuscript drafting, performed image post-processing, and statistical analysis. HL prepared the radiology images. JY was responsible for the revision of the manuscript for important intellectual content. BW provided the cases. All authors issued final approval for the version to be submitted.

## Funding

This study was approved by Key Laboratory of Functional Molecular Imaging of Tumor and Interventional Diagnosis and Treatment of Shaoxing City.

## Conflict of interest

The authors declare that the research was conducted in the absence of any commercial or financial relationships that could be construed as a potential conflict of interest.

## Publisher's note

All claims expressed in this article are solely those of the authors and do not necessarily represent those of their affiliated organizations, or those of the publisher, the editors and the reviewers. Any product that may be evaluated in this article, or claim that may be made by its manufacturer, is not guaranteed or endorsed by the publisher.
